# Saphenous Vein–Preserving Inguinal Lymph Node Dissection: A Stepwise Technical Approach

**DOI:** 10.1002/ccr3.71712

**Published:** 2025-12-29

**Authors:** Elise Lupon, Ioana Ivan, Olivier Camuzard, Dimitri Gangloff

**Affiliations:** ^1^ Department of Plastic and Reconstructive Surgery Institut Universitaire Locomoteur et du Sport, University Côte D'azur, Pasteur 2 Hospital Nice France; ^2^ Université Côte D'azur, CNRS, LP2M Nice France; ^3^ Department of Plastic and Reconstructive Surgery Institut Universitaire Du Cancer Toulouse Oncopole Toulouse France

**Keywords:** dermatology, inguinal lymph node dissection, melanoma, oncology, surgery

## Abstract

Preserving the great saphenous vein during inguinal lymph node dissection maintains venous outflow and skin drainage while allowing complete oncologic clearance.

## Highlights

- Saphenous vein preservation is a simple technical modification during inguinal lymphadenectomy.
- Medial‐to‐lateral stepwise dissection allows vein and arch preservation while ensuring oncologic clearance.
- Passing lymphatic tissue beneath the vein maintains venous drainage.
- Technique is easily reproducible and integrates into conservative groin dissection strategies.

## 1 Clinical Question

What simple technical modification can be used during inguinal lymph node dissection to maintain venous drainage?

## 2 Answer

Saphenous vein preservation is a straightforward technical modification that maintains an important venous outflow pathway during inguinal lymphadenectomy (Video 1). The technique begins with medial identification of the vein and careful clearance of lymphoadipose tissue while keeping the saphenous vein and arch intact. Passing lymphatic tissue beneath the vein maintains oncologic adequacy without disrupting venous drainage. This stepwise approach illustrates a practical refinement of traditional groin dissection [1, 2] (Figure S1).

**VIDEO 1:** Video demonstration of a saphenous vein–preserving inguinal lymph node dissection. Video content can be viewed at https://onlinelibrary.wiley.com/doi/10.1002/ccr3.71712.

## Author Contributions

**Elise Lupon:** conceptualization, data curation, formal analysis, funding acquisition, investigation, methodology, project administration, software, visualization, writing – original draft, writing – review and editing. **Ioana Ivan:** data curation, project administration, resources, writing – review and editing. **Olivier Camuzard:** funding acquisition, project administration, supervision, writing – review and editing. **Dimitri Gangloff:** conceptualization, project administration, resources, supervision, validation, visualization, writing – review and editing.

## Funding

The authors have nothing to report.

## Consent

Written informed consent was obtained from the patient for publication of this case and accompanying images/video.

## Conflicts of Interest

The authors declare no conflicts of interest.

## Supporting information


**Figure S1:** Still frame from the supplementary video demonstrating the preservation of the great saphenous vein during inguinal lymph node dissection. The femoral vein with its preserved great saphenous vein lies medially, while the femoral artery is seen laterally.

## Data Availability

No datasets were generated or analyzed during the current study. The video material supporting this publication is included along with this article.
