# Supplementary figures and images for: Saphenous Vein–Preserving Inguinal Lymph Node Dissection: A Stepwise Technical Approach
Source: Clin Case Rep. 2025 Dec 29;14(1):e71712. doi: 10.1002/ccr3.71712 (PMC12998247; doi:10.1002/ccr3.71712)

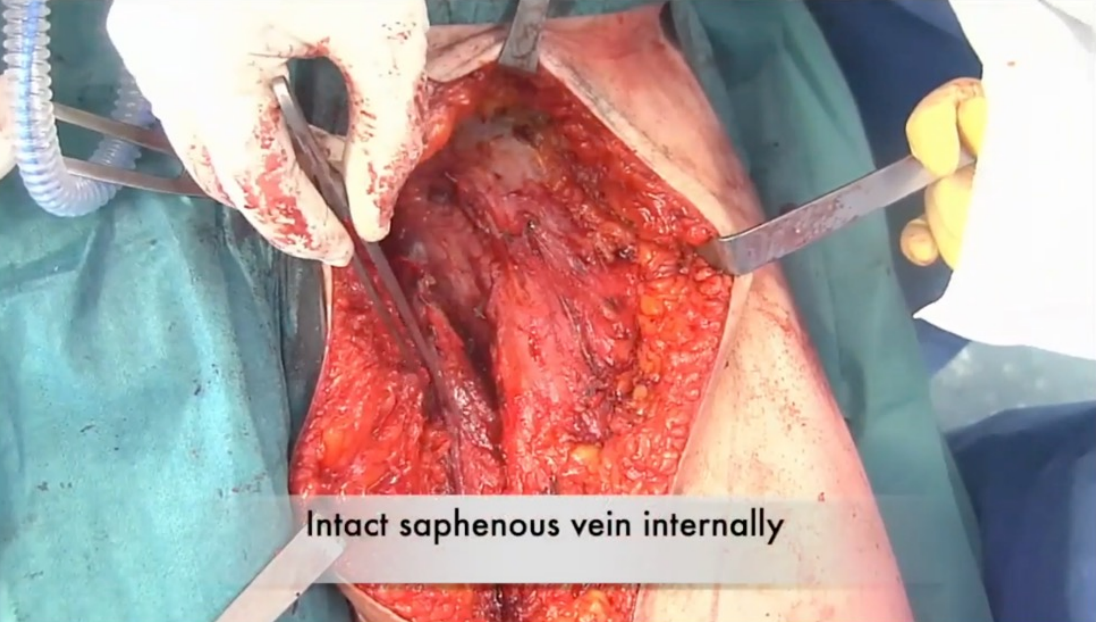

Intact saphenous vein internally

Supplement: Supplementary file 2 — Figure S1: Still frame from the supplementary video demonstrating the preservation of the great saphenous vein during inguinal lymph node dissection. The femoral vein with its preserved great saphenous vein lies medially, while the femoral artery is seen laterally. [file CCR3-14-e71712-s001.pdf]
